# Large-Scale Counting and Localization of Pineapple Inflorescence Through Deep Density-Estimation

**DOI:** 10.3389/fpls.2020.599705

**Published:** 2021-01-28

**Authors:** Jennifer Hobbs, Prajwal Prakash, Robert Paull, Harutyun Hovhannisyan, Bernard Markowicz, Greg Rose

**Affiliations:** ^1^IntelinAir, Inc., Champaign, IL, United States; ^2^Department of Electrical Engineering, Columbia University, New York, NY, United States; ^3^Department of Tropical Plant and Soil Sciences, University of Hawaii at Manoa, Honolulu, HI, United States

**Keywords:** deep learning-artificial neural network (DL-ANN), active learning, pineapple, computer vision, remote sensing-GIS, weakly supervised, counting, density estimation

## Abstract

Natural flowering affects fruit development and quality, and impacts the harvest of specialty plants like pineapple. Pineapple growers use chemicals to induce flowering so that most plants within a field produce fruit of high quality that is ready to harvest at the same time. Since pineapple is hand-harvested, the ability to harvest all of the fruit of a field in a single pass is critical to reduce field losses, costs, and waste, and to maximize efficiency. Traditionally, due to high planting densities, pineapple growers have been limited to gathering crop intelligence through manual inspection around the edges of the field, giving them only a limited view of their crop's status. Through the advances in remote sensing and computer vision, we can enable the regular inspection of the field and automated inflorescence counting enabling growers to optimize their management practices. Our work uses a deep learning-based density estimation approach to count the number of flowering pineapple plants in a field with a test MAE of 11.5 and MAPD of 6.37%. Notably, the computational complexity of this method does not depend on the number of plants present and therefore efficiently scale to easily detect over a 1.6 million flowering plants in a field. We further embed this approach in an active learning framework for continual learning and model improvement.

## 1. Introduction

Specialty crops, such as pineapple (*Ananas comosus* L.), present unique challenges and require sophisticated approaches to maximize productivity. Growers of large area crops such as corn or soybean have access to GPS-based yield maps and precisely apply inputs such as fertilizer and water considering field variability. Specialty crop growers lack access to these data as their crops tend to be hand-harvested. Because of this, specialty growers have been at a disadvantage, having to make decisions without this level of insight.

**Figure 1:**
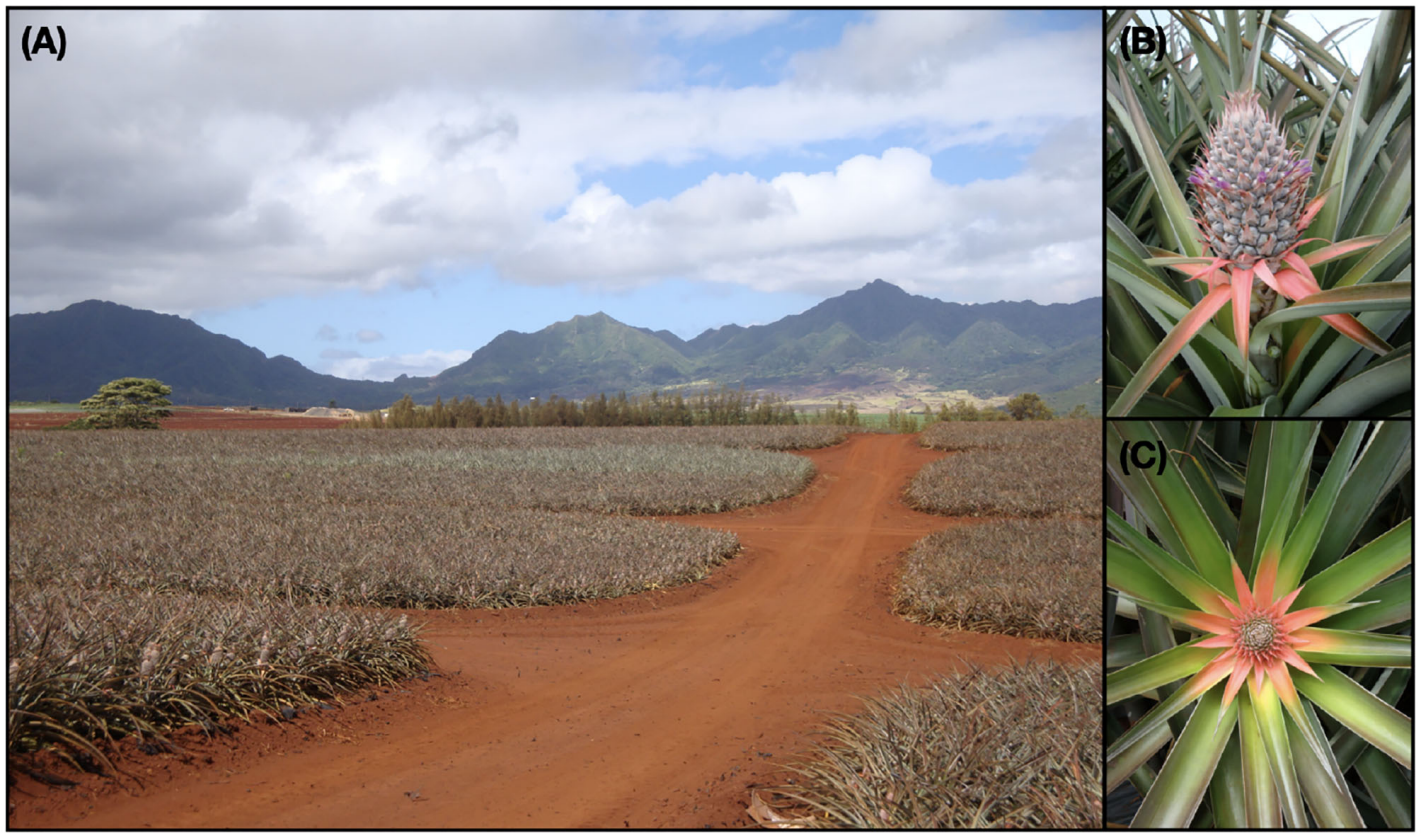
**(A)** A ground-level view of a pineapple field shows the large number and high density of plants (25,000–30,000 plants per acre) which makes inspecting the interiors of the blocks difficult. **(B)** An oblique view of a pineapple plant at the mid-flowering stage and early fruit growth stage tucked away among the leaves. **(C)** The top-down view shows the characteristic red center of a plant at an early stage of flowering called the “red-bud early cone” stage.

Growers of these high-value crops make a number of key decisions in every growing cycle. For pineapple, data supporting these decisions are generally limited to visual ground observations. But these observations are from the periphery where spatial and temporal variability, stage of growth, and development cannot be determined or quantified across the entire field. Walking through the field is difficult as the plants grow very close together: 30,000 plants per acre (Figure 1). This lack of complete, real-time information about field conditions can lead to poor decisions resulting in too little or too much water, fertilization, pesticides, and growth regulators, or poor planning and scheduling of planting and harvest resources, including equipment and labor.

The natural flowering of pineapple affects fruit development and quality, and impacts harvest (Py et al., 1987; Bartholomew et al., 2002; Zhang and Kovacs, 2012; Sanewski et al., 2018). Pineapple growers use chemicals which produce ethylene (Ethephon) to induce flowering so that most plants within a field produce fruit of high quality ready to harvest at about the same time (Paull and Duarte, 2011; Bartholomew, 2013). The ideal situation would be for a grower to harvest the entire field in one pass when there is little variation in flowering, significantly increasing productivity and eliminating the cost of additional harvests.

Advances in aerial imagery collection (e.g., drones, UAVs) and remote sensing allow the grower insight into his field that was previously unattainable (Jung-Rothenhaeusler et al., 2014). UAVs have been deployed in large scale pineapple operations to reduce erosion and manage crop fertilization programs (Jung-Rothenhaeusler et al., 2014). However, their application to other aspects of managing pineapple production such as counting and identifying flowering pineapple plants from such imagery remains challenging because: 1. Pineapple inflorescence dramatically change in appearance (both size and color) as they develop and mature (Bartholomew et al., 2002; Zhang H. et al., 2016) 2. The global appearance of fields varies significantly due to lighting, shadowing, and other illumination differences. 3. A single field may have 1–2 million plants; methods where computational efficiency scales with the number of entities would be prohibitive at scale.

**Figure 2:**
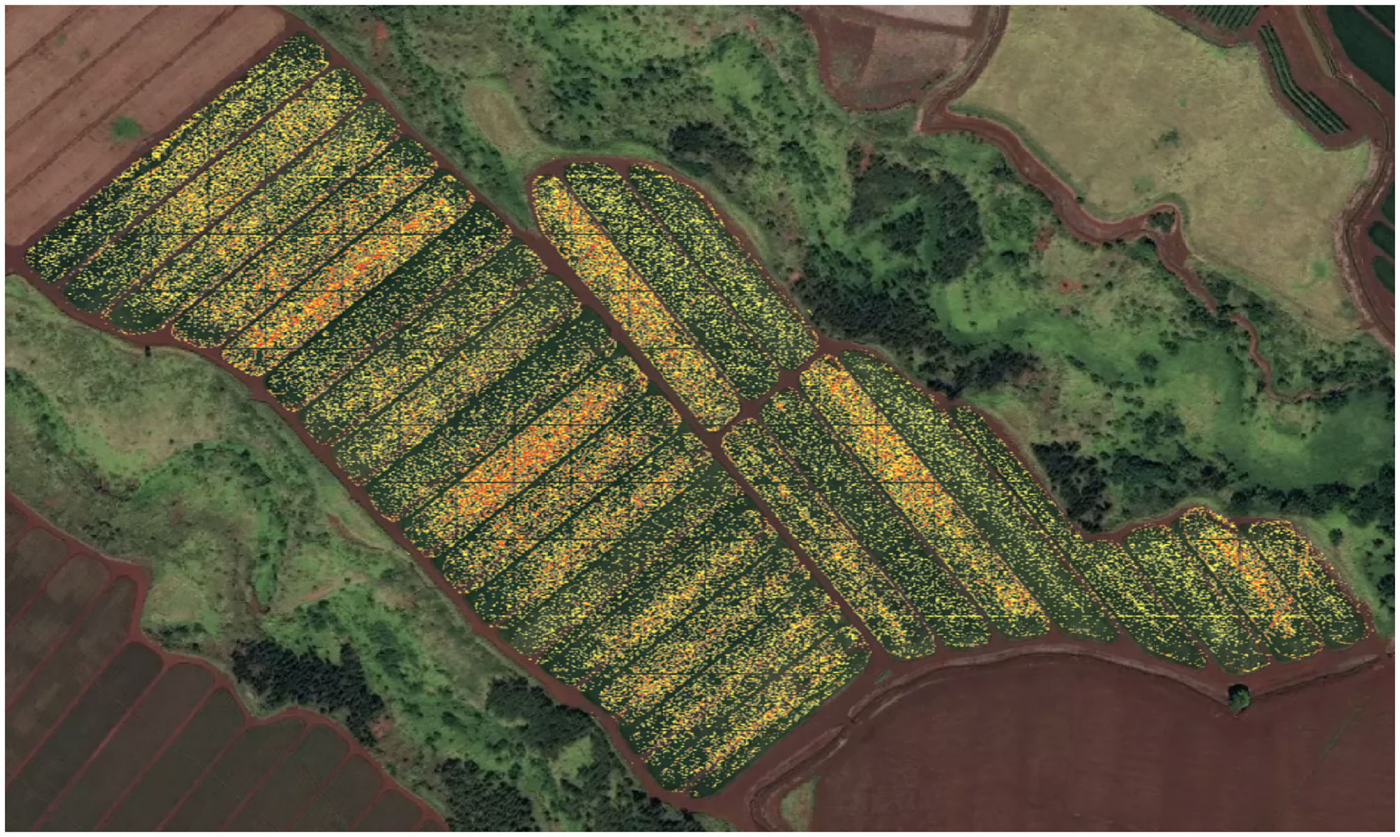
Our model identifies the density of flowering across multiple blocks of a pineapple field. The flowering density is depicted on a spectrum from low (yellow) to high (red) and regions of no-flowering are shown as transparent (i.e., green from imagery shows through). A single, example field of 61.5 acres has over 1.6 million plants. This field has blocks at all stages of flowering and early fruit development. On the right hand side of the field, the plants are at the early stages of flowering with many still vegetative. The grid-like pattern observed across the image corresponds to the access roads (16 feet wide) surrounding each block with each block being 126 feet wide and of varying length; the model has accurately identified these as non-flowering areas. Several of the blocks are seen to have lower flowering density as they were just beginning to flower and still largely vegetative as confirmed in ground-inspections by horticulturalists.

Our work leverages the advances of deep learning to automatically count and localize flowering pineapple plants, which may be in the millions for a single field (Figure 2). We use a counting-by-density-estimation approach to produce a density map of pineapple inflorescence across the field. This approach determines the density distribution of fruits across all regions of the field and identifies areas which are ready for harvest or delayed in development. Our approach produces results occasionally better than the human annotations.

Additionally, we embed this density-estimation framework in an active learning paradigm. After the density-inference is complete for a new image, we extract discrete locations of each inflorescence using a peak finding algorithm. These points are sent to human annotators for corrections and the model is retrained on the new data; this enables the model performance to improve as it sees more and more data while reducing the burden on human annotators. While active learning has been previously applied to plant counting tasks in a counting-by-detection paradigm (Ghosal et al., 2019), our novel approach extracts discrete peaks that can be corrected by annotators while maintaining the computational advantages of the counting-by-density-estimation approach.

Finally, we demonstrate the usefulness and qualitative performance of our approach through field inspections. We see that the algorithm performs well across all stages of flowering, even though the appearance of the inflorescence in each stage varies. Our algorithm successfully identifies areas of stunted flowering occurring naturally or due to other circumstances (e.g., irrigation, fertilization, spraying for flower induction). The inspections also showed that there was about 1.4% plants missing in a row and about 12% of plants had fruit that were small, on short fruit stems or covered by leaves from adjoining plants and hence not easily discernible from above.

## 2. Related Work

### 2.1. Counting Methods

Work in the area of dense-crowd-counting has inspired much of our current work (Loy et al., 2013; Sindagi and Patel, 2018). Within the broader domain of (entity) counting, approaches fall under one of three categories: counting by detection, counting by regression, and counting by density estimation (Sindagi and Patel, 2018).

Detection-based approaches are most applicable when the entities are large and well-separated, occlusions are limited, and the number of entities is small. These may take the form of sliding-window approaches that detect all or part of the entity in question (Li et al., 2008; Dollar et al., 2011) and sum the detections over the entire image. With the success of deep learning, many of these traditional approaches have been replaced with neural network-based detection and segmentation algorithms (Ren et al., 2015; Redmon et al., 2016; He et al., 2017), but these new methods still seek to solve the counting problem through the precise localization of all desired entities in the image. Key drawbacks to these methods are they tend to be computationally heavy, the time complexity often scales with the number of entities present, they often have an upper-limit of detectable entities before encountering memory issues, and they tend to struggle as occlusion becomes more pronounced or the entities become small. Additionally, detection methods, like Faster R-CNN (Ren et al., 2015) and YOLO (Redmon et al., 2016) require bounding box annotations and Mask R-CNN (He et al., 2017) further requires dense instance mask annotations, all of which are extremely time consuming to acquire.

In contrast, counting by regression approaches eliminate the need to determine locations of each entity and seek only to determine the number of entities present (Chan and Vasconcelos, 2009; Ryan et al., 2009; Chen et al., 2012); these approaches also have benefited tremendously from deep learning-based architectures (Wang et al., 2015). However, these methods when used on their own provide only the total count, without any information as to how the entities are distributed across the image.

Density estimation approaches have proven very successful (Lempitsky and Zisserman, 2010; Pham et al., 2015; Xu and Qiu, 2016) especially when combined with deep architectures (Boominathan et al., 2016; Onoro-Rubio and López-Sastre, 2016; Zhang Y. et al., 2016; Sam et al., 2017, 2019) when we desire localization in addition to a final count. Many of these leverage fully convolutional neural networks (FCNs) to predict a density (Xie et al., 2018; Ma et al., 2019) across the image; this density can be integrated to provide the count over a region. Furthermore, these methods tend to outperform detection-based methods in highly occluded scenarios. They also require only simple point-annotations which can be acquired far more quickly than the bounding-box or instance-mask annotations needed by detection methods. Additionally, because the output density map is itself a single-channel image, not a collection of bounding boxes, the computational complexity is independent of the number of entities present. Our approach follows these methods as inflorescence may be occluded by other portions of the plant, and the number of inflorescence in a given image could be extremely large.

### 2.2. Active Learning

Deep learning approaches require a large amount of labeled data to maximize their performance and therefore a significant demand can be put on human annotators to gather such data. To offset these demands, significant work has been done in weakly, semi, and self-supervised learning approaches (Rosenberg et al., 2005; Zhu, 2005; Ratner et al., 2019; Xie et al., 2020). Most relevant to the present work are the weakly supervised approaches that incrementally train a model on a selection of data, correct any erroneous predictions using a human annotator or “oracle,” and then retrain the model on the larger set of correctly annotated data (Li et al., 2013; Zhou et al., 2016). Active Learning is a subset of this domain which further explores the optimal selection of data for training (Settles et al., 2008; Settles, 2009; Huang et al., 2010). Many of these approaches rely on finding disagreement sets between different models trained for the same task (Dagan and Engelson, 1995; McCallum and Nigam, 1998) while others seek to find regions of uncertainty directly (Cohn et al., 1994) in the input space. The goal of our work is not around proposing a new or better query strategy, but to demonstrate how an active learning approach can improve results and reduce annotation cost in this domain.

### 2.3. Applications in Agriculture

Both traditional computer vision and deep learning-based approaches have been used for a variety of counting-based agricultural applications. The work of Guo et al. (2018) and Malambo et al. (2019) used detection-based techniques to detect sorghum heads in a field. Similarly, Gené-Mola et al. (2020) used Mask-RCNN to fully identify and segment apples on trees in an orchard. To count palm trees from UAV imagery, Li et al. (2017) used a CNN-based detection approach. Very recently, Osco et al. (2020) used an approach very similar to ours to count the number of citrus trees in a grove. Where they sought to count every tree present, in our work we seek to count only those plants who have begun to flower.

Particularly related to our work is Ghosal et al. (2019) who used a RetinaNet-based approach (Lin et al., 2017) to simultaneously regress the total count and individual bounding boxes of sorghum heads. This network was embedded into their “automated annotation protocol” (i.e. active learning system; Settles, 2009). We similarly embed our network into an active learning paradigm to enable continual learning. However, our counting approach is based on density-estimation approaches and does not rely on bounding box detections as in the above work.

## 3. Materials and Methods

### 3.1. Data

**Figure 3:**
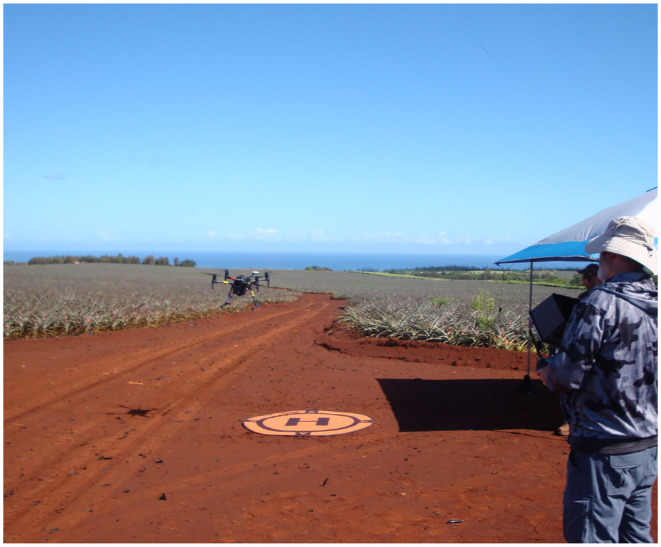
A photo of the drone preparing to begin its imagery collection flight over the pineapple fields, with the Pacific Ocean in the distance on the north side of Oahu, Hawaii.

We acquired raw imagery via a DJI Matrice 210 drone equipped with a DJI X3 three band (RGB) camera flown at a height of 200 ft above the pineapple fields (Figure 3). Individual images were stitched together using a third party system (Pix4Dmapper) to produce a single large-scale image for each block. During the stitching process, orthorectification is performed using the RGB image and a digital elevation model (DEM) of the field (Gao et al., 2009; Laliberte et al., 2010).

From this full dataset we randomly sampled 866 patches (512 × 512) across flights over 12 blocks from three fields for annotations. Annotators marked the center of each inflorescence with a point-label, producing 76,659 total point annotations. The data was split such that 650 patches for training and 130 patches for validation were sampled from multiple blocks belonging to an initial set of fields and 106 patches for testing were sampled from blocks belonging to an entirely different set of fields. That is, no field which appeared in the test set appeared in either the training or validation sets.

For training, we performed the following augmentation steps: the original sample (and label) was rotated by a random angle and randomly cropped to 256 × 256. For testing and validation, the original 512 × 512 patches were split into four non-overlapping 256 × 256 images.

### 3.2. Density Estimation

To produce the target density map, the point labels generated by annotation were blurred using a two-dimensional isotropic Gaussian filter. That is, given an image *I* with pixels ***x***_*m*_ annotated with points $z_{n}=\{z_{1},\ldots ,z_{N}\}|z_{i}\in R^{2}$ where *N* is the total number of points annotated in that image, we define the *ground truth* density map **D** to be a kernel density estimate given by:

(1) $$\begin{array}{r} D\left(x_{m}\right)\overset{\text{def}}{=}\overset{N}{\underset{n=1}{\sum }}N\left(x_{m};z_{n},\sigma^{2}1_{2\times 2}\right) \end{array}$$

(2) $$\begin{array}{r} =\overset{N}{\underset{n=1}{\sum }}\frac{1}{\sqrt{2\pi \sigma }}\exp\left(-\frac{∥x_{m}-z_{n}{∥}_{2}^{2}}{2\sigma^{2}}\right) \end{array}$$

We explored values in [1, 2, 6, 10, 20] for σ, the standard deviation of the Gaussian kernel, and found that σ = 6 provided the best results both in terms of MSE as well as steps needed for convergence.

We used the mean squared error (MSE) between the target and predicted density maps $\hat{D}\left(x_{m}\right)$ as our loss function and is given by

(3) $$\begin{array}{r} MSE=\frac{1}{M}\overset{M}{\underset{k=1}{\sum }}\|D\left(x_{m}\right)-\hat{D}\left(x_{m}\right){\|}_{2}^{2}. \end{array}$$

Adam Optimizer was used with a learning rate of 0.001, β_1_ = 0.9, β_2_ = 0.99, and weight decay of 1*e*^−5^. The model was trained with a batch size of 20 on a machine equipped with an NVIDIA Titan RTX for up to 1,000 epochs; the final model was halted using early stopping after 30 steps. In early work, we used a machine with a Tesla P4 with a batch size of 10. The optimal model on this hardware was not reached until 630 epochs (which is why the maximum allowed epochs was set to 1,000) and did not yield as good of results as the final model trained on the Titan RTX.

**Figure 4:**
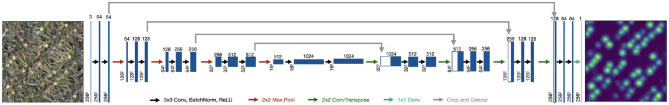
Our architecture follows the encoder-decoder structure of U-net where the input is an RGB image and the output is a density map.

Our model used the fully-convolutional encoder-decoder structure of U-net (Ronneberger et al., 2015), taking in the 3 (RGB) input channels and producing a single-channel output corresponding to the inforescence density (Figure 4). Each convolutional block consisted of 3x3 convolution followed by batch normalization (Ioffe and Szegedy, 2015) and a ReLU nonlinearity. Max Pooling with a 2 × 2 kernel with a stride of 2 was used in the encoder after every two convolutional blocks. In the decoder, we used a 2 × 2 transposed convolution for upsampling. We used *same* padding throughout.

The final layer consists of a 1D convolution followed by ReLU activation: this ensures that every point in output layer is positive, which is required by our density prediction task. Note, that the output density is *not* required to be [0, 1], but only positive; if many inflorescences are located closely together, their densities could add to >1 in some places. In practice, we did not see this occur and therefore a final sigmoid activation could be used in place of the ReLU to enforce a range of [0, 1]. However, we found that the final ReLU activation outperforms these alternatives.

#### 3.2.1. Total Count

The output of the U-net is a single channel density map of the flowering plants across the field. To get the total count of inflorescence ${\hat{T}}_{c}$ in a particular region, in this case the sample window, we integrated over the density map to produce the final count. That is, ${\hat{T}}_{c}=\int D\left(x_{m}\right)dx$. Note that *dx* corresponds to the spatial window captured by a single pixel and therefore in practice this equations to taking the sum of the prediction matrix.

### 3.3. Weak Supervision and Active Learning

#### 3.3.1. Weakly Supervised Annotation Framework

**Figure 5:**
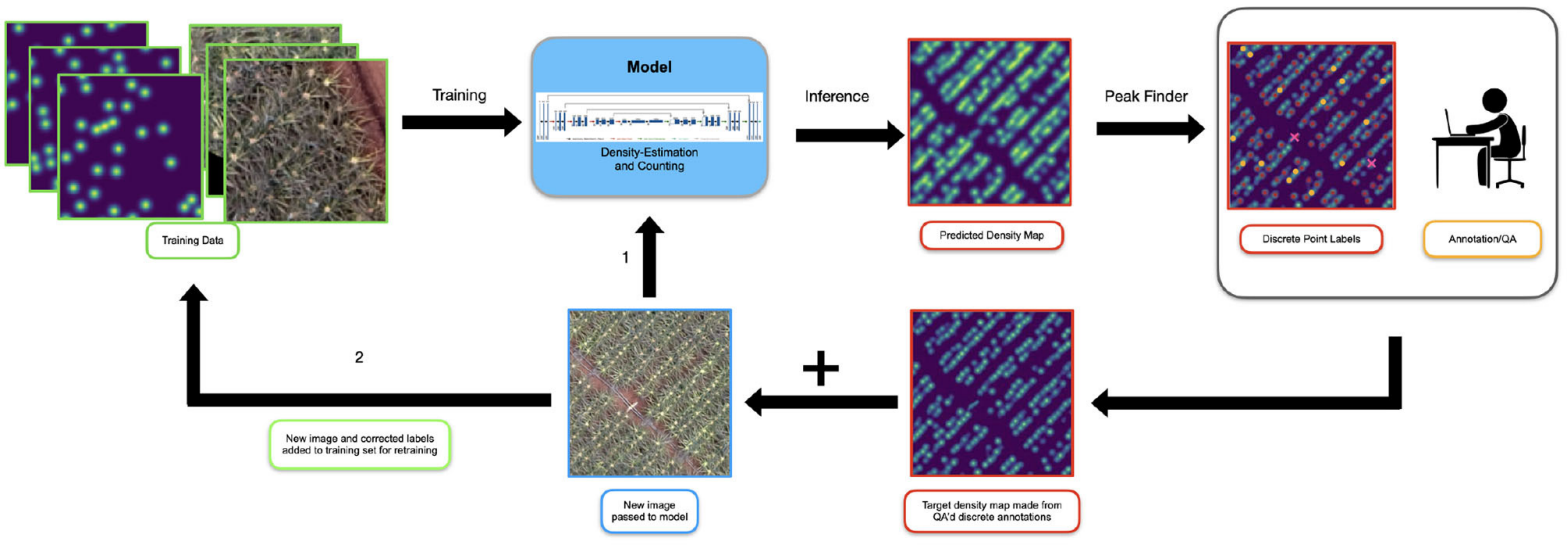
The setup for our active learning system. Initially the model is trained on images which have been annotated from scratch. When new data is initially presented to the model (1), it is passed directly in for inference. The model generates the predicted density map and the peak finding algorithm is used to extract discrete locations of inflorescences. A human annotator or “oracle” reviews and corrects the discrete points and a target density map is created for the new sample. Note, the “predicted” points shown here has been made artificially poor to emphasize the actions of the annotator. This map and the original image are added to the training set (2) and the model is retrained.

We used a weakly supervised approach to continually feed more (annotated) data to the model. Figure 5 shows an overview of this approach.

As discussed previously, the model was trained on an initial training dataset. When new (unseen) data becomes available, it is passed directly to the model for inference; the U-net produces a predicted density map for that image.

Commonly in an active learning paradigm, the model output is cleaned up directly by human annotators and fed back into the model for retraining. However, cleaning up the density map directly is a challenging annotation task because the “location” of the inflorescence is non-uniformly spread over a set of pixels. Where inflorescence distributions overlap, it is unclear how the density map should be appropriately altered; inconsistency and ambiguity from the annotators would degrade, not enhance model performance.

To overcome this, we developed a procedure to extract discrete locations of points that can be submitted for re-annotation from this final density map. We first threshold the image so regions of low density, below γ, are removed. Next, we use a 2D local-max finding algorithm common to most image processing toolkits to identify peaks requiring a minimum distance of δ between peaks. We found that *γ* = 0.05 and *δ* = 4 work well in practice although these values can be dynamically changed in the annotation interface to best support the annotation process. Note that because of the filtering applied during this process, the sum over these peaks ${\hat{T}}_{d}$ will *always* be *less* than the overall predicted count obtained by integrating over the predicted density map ${\hat{T}}_{c}$. This is not problematic and in fact, we found anecdotally that annotators (aka. the “oracle”) seem to be more efficient and accurate at adding missed detections as opposed to deleting false positives.

Next the set of discrete point annotations, after having been corrected by the oracle, is smoothed with the same Gaussian filter used on the initial data to create a new target (i.e., ground-truth) density map label. This new label along with the image is added to the training set for retraining. The validation set is left unchanged.

Retraining occurs whenever a “sufficient” amount of new annotated data is acquired: sufficiency is usually determined by operational constraints such as cost or compute time. After the model is retrained, if it outperforms the previous model on the validation set, it is promoted to the current version and used for subsequent inference. This process is repeated as desired.

#### 3.3.2. Active Learning

In the passive weakly supervised approach, new samples are fed to the model randomly. However, we also seek to minimize annotator burden and maximize the efficiency of the model training process by prioritizing the most “useful” and informative samples for annotation and retraining. Therefore in the Active Learning approach, we prioritize samples in the following manner:

1. The total count ${\hat{T}}_{c}$ for a given (new) sample is computed by integrating over the predicted density map.
2. The peak-finding algorithm is applied to identify discrete locations of flowers. The number of discrete points is ${\hat{T}}_{d}$.
3. The absolute difference $\text{CountDiff}=\left({\hat{T}}_{c}-{\hat{T}}_{d}\right)$ is computed.
4. Samples are ranked according to *CountDiff* and the samples with the greatest differences are prioritized for annotation and retraining.

#### 3.3.3. Impact of Data Quantity and Learning Strategy

In practice, new data will be passed to the model during passive and active learning. However, to quantify the impact of more data on the model performance which the active learning system affords, we conducted an experiment in which we incrementally trained the original model on growing amounts of the original training set.

In the following experiments, the validation and test sets were identical to before. Only the subset of training data which the model was shown at each step was varied. For clarity, we denote the set of training data which was not currently being used at that step of training “the training (data) pool.”

The model was initially trained on a 50 samples of training data and validated against the full validation set. Inference was run on the test set and the performance was recorded. Additional samples were selected from the training pool and added to the initial 50 samples according to the following procedure:

1. Inference was run on the test set to record performance for that amount of training data.
2. Inference was run against the training data pool.
3. *CountDiff* was computed for all samples in the training data pool.
4. Those samples with the largest value were added to the training set for the next round of training.
5. The model was retrained.
6. These steps were repeated until all data from the training data pool had been added to the training set.

We added data and retrained at levels of [50, 100, 250, 500, 650] samples. Results are shown in section 4.2.

### 3.4. Ground Inspections

To provide ground-level verification of the model's output and to demonstrate how this application could potentially be incorporated into one's management practices, we inferenced and conducted ground inspections of a block.

After model training, validation, and testing was complete, we ran inference on a completely unseen block; this block belonged to the same field and was under the same management as those areas used for training-validation-testing, but was not previously shown to the model. In particular, pineapples in the field were induced to flower when the plants were large enough by spraying with a chemical (Ethephon) that breaks down to release ethylene; ethylene is the natural inducer of flowering in Bromeliads of which pineapple is a member. A density map for the entire block was constructed to enable clear visualization of the distribution of inflorescences across the block and easy identification of any areas which may be exhibiting stunted development or early inflorescence.

Three horticulturalists familiar with pineapple flowering evaluated inflorescence in that block. Inflorescence number and their visibility were counted in a 50 feet bed that has two rows of pineapple plants; this evaluation was repeated four times. Qualitative evaluation was carried out by walking around the field block's perimeter and estimating the stage of flowering as red bud, early or late cone and early mid, late flowering and dry petal stage or early fruit development of each block in a field. Red Bud is the first noticeable stage of inflorescence development, with the cone stage being the later stage of inflorescence development before flowers begin to open from the base of the inflorescence cone.

## 4. Results

### 4.1. Density Estimation

#### 4.1.1. Model Performance

**Figure 6:**
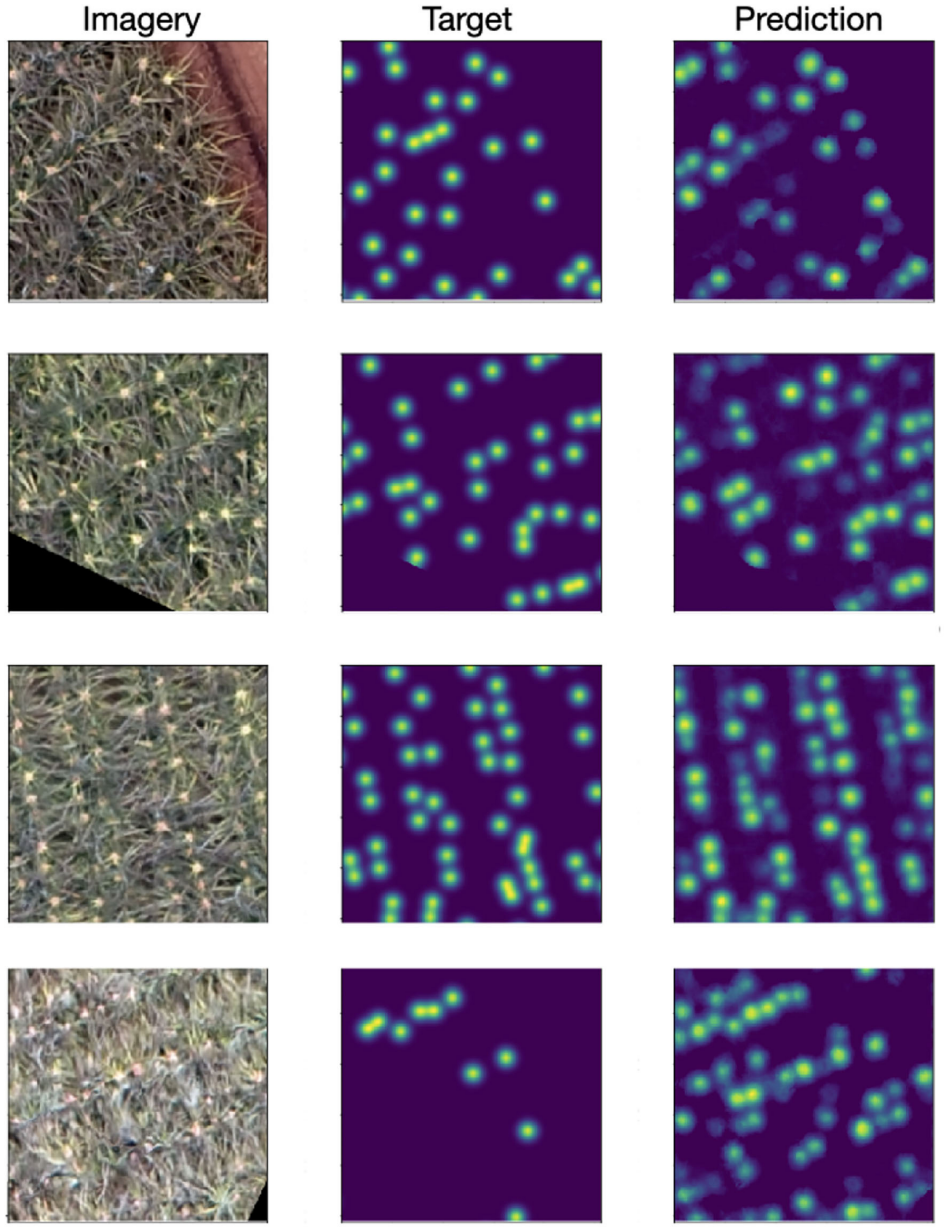
**(Left)** Input RGB image. **(Middle)** Target density maps generated from the human point annotations and smoothed with a Gaussian kernel with σ = 6. **(Right)** Predicted density map. Particularly when the inflorescence are less well defined, the model can be seen to outperform the human annotations (bottom row).

Results from our approach are shown in Figure 6. The per-pixel MSE validation loss was 0.0033 and the test loss was 0.0038. Qualitatively we see the predicted density maps closely resemble the target maps. In certain cases, particularly when the inflorescences are redder in appearance (corresponding to earlier stages of flowering), the outputs of the model occasionally appear more correct than the initial human annotations.

#### 4.1.2. Total Count

**Figure 7:**
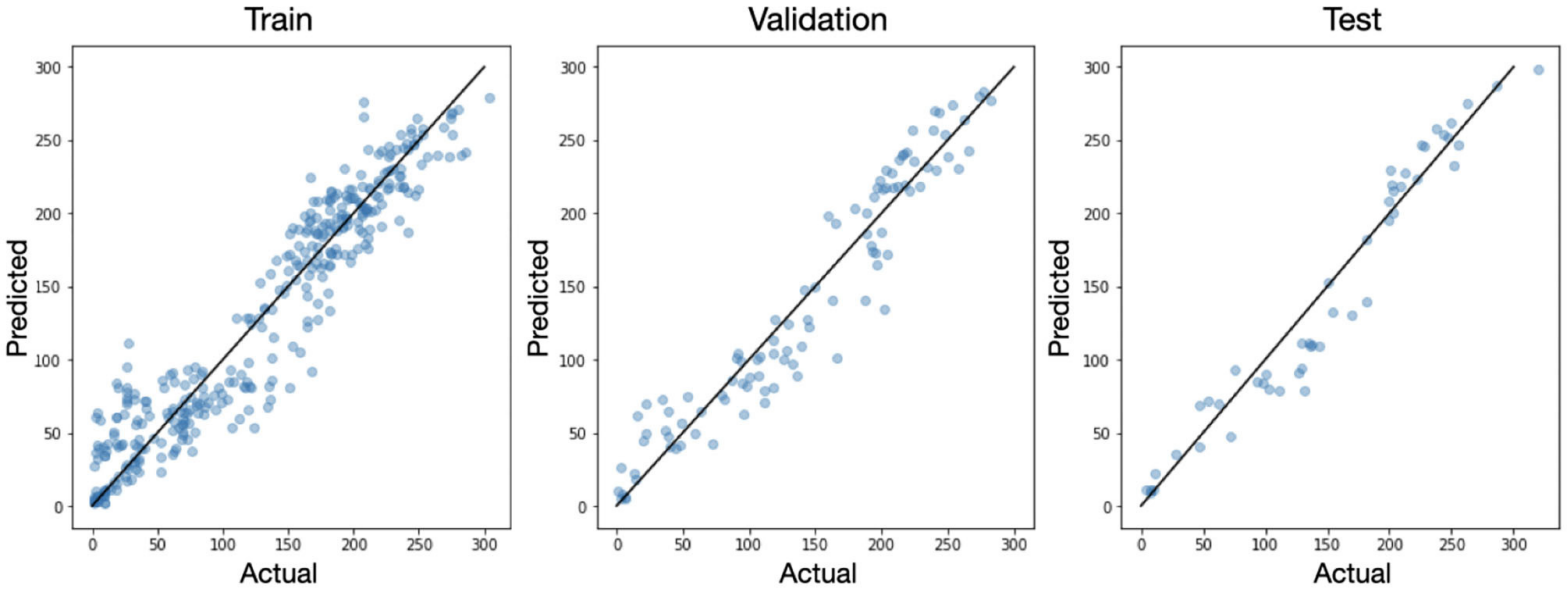
The actual vs. predicted number of inflorescence shown for each sample in the training, validation, and test sets. The black line corresponds to x=y.

Integration of the predicted density maps over the entire image provides us with a prediction of the total number of inflorescence. For each original image we compared the actual number of flowers to the number predicted by the model as seen in Figure 7. Because the U-net is a fully convolutional network, it is amenable to figures of variable sizes so long as the pooling operations result in integer dimensions. So for this analysis, we inferenced the original 512 × 512 images without any augmentation (i.e., rotation or cropping) in the training, validation, and test sets. We see that in all three splits, the data falls close to the x=y line with a mean absolute error (MAE) of 11.5 and mean absolute percent deviation (MAPD) of 6.37% on the test set.

#### 4.1.3. Computational Efficiency

The computational efficiency of this approach offers key advantages. Inference speed is 0.04 sec/sample on a single P4 GPU and under 0.0039 sec/sample on a single Titan RTX. Especially with appropriate compilation steps which would even further increase efficiency, this speed would enable the model to be run in real-time, potentially allowing for on-the-fly decision making.

### 4.2. Impact of Data Quantity on Performance

**Figure 8:**
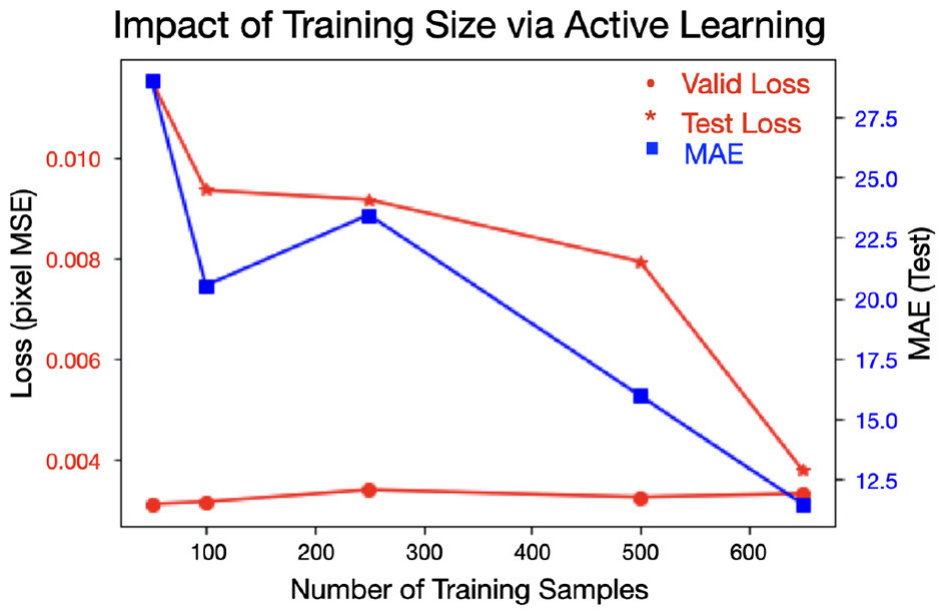
Increasing the amount of (labeled) training data in a smart fashion decreases test loss as well as the MAE on the test set. The validation loss slightly increases as more data is added, suggesting less over-fitting is occurring as more data is added.

Figure 8 shows the impact of enlarging the dataset via our active learning approach. Recall that the validation set was in the same domain as the training set (i.e., different samples from the same fields) while the test set was out-of-domain (i.e., samples from a completely unseen field). We see that as more data was added, the test loss (red stars) decreased, as we hoped. Additionally, the MAE on the test set (generally) improved. The validation loss slightly increased, but not significantly. This may suggest that as the quantity of data is increased, the model is less likely to (over)fit to the in-domain samples of the training and validation sets, while the generalizability (as seen in the test performance) improves.

### 4.3. Qualitative Analysis and Ground Inspections

Using the final supervised model, we ran inference on a new field (independent of the train, validation, or test set) to generate its density map. Horticulturists then inspected the field, particularly focusing on areas which the model deemed to be low-density.

**Figure 9:**
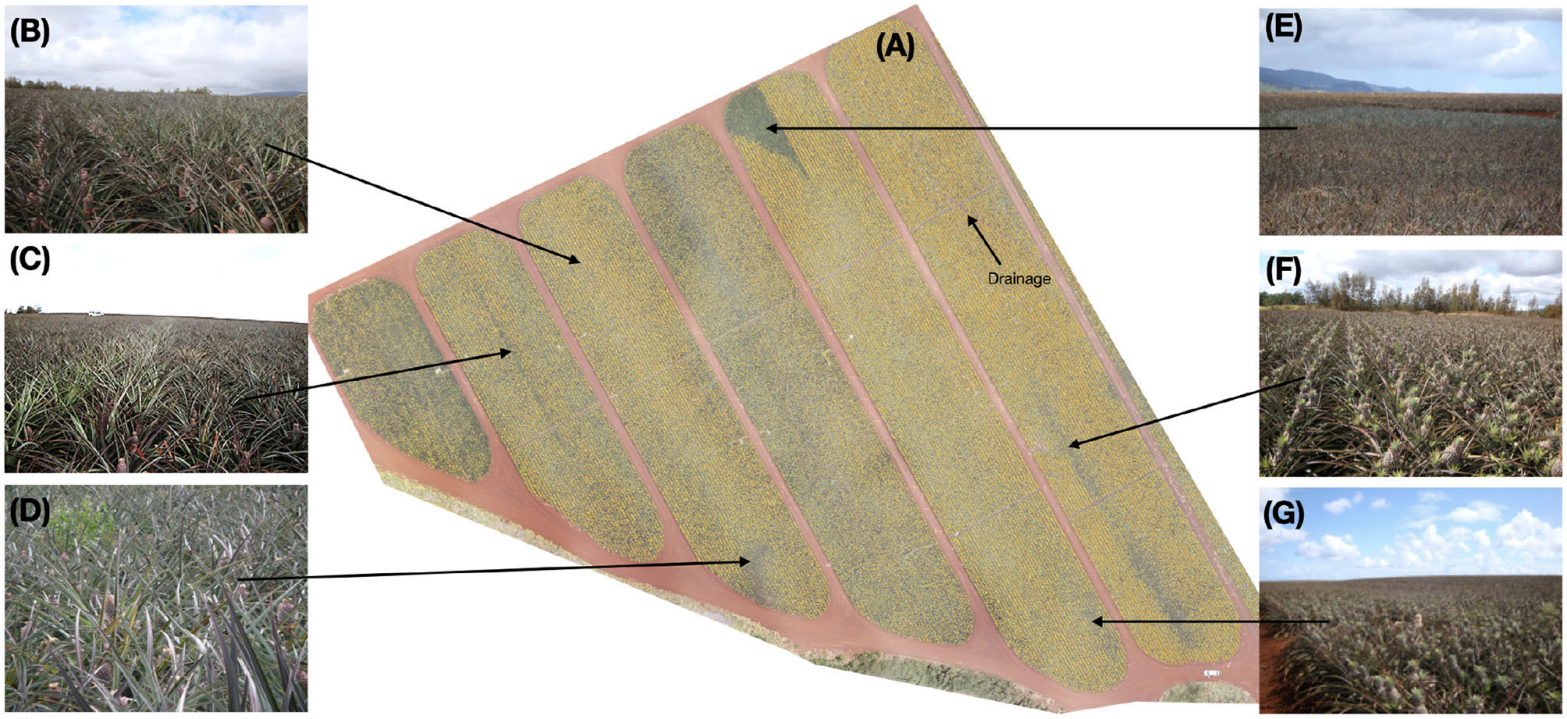
**(A)** Inference was run on a previously unseen block and the density map is overlaid on the imagery (transparent: none, low: yellow, red: high). Variation across the block is clearly visible. In some cases, these come from known field-issues such as drainage or irrigation lines. Others, however, require inspection from horticulturist to determine the source. **(B)** This region of the field has mostly normal flowering. **(C)** Failed forcing is seen in the middle bed of this block and extends nearly half the length in the block where the amount of flowering (62% flowering) in that bed of two rows is lower than the surrounding beds (98% flowering). **(D)** The model predicted slightly lower-than-average density in this region, however, inspection showed the ground-truth density was normal. Plants in this area appear to be shorter than average; this can cause the young fruit to be more easily obscured from the imagery. **(E)** A vegetative region of the field is clearly visible from the density map. **(F,G)** Mosaicking issues resulting in fuzzy imagery resulted in the model predicting lower-than-average density in these areas when ground-truth inspection revealed otherwise. Best viewed electronically.

Figure 9 shows the inspected blocks, predicted density map, and several ground-level images taken during inspection. The density map (Figure 9A) draws your attention to key areas on the field. Interesting features of the plot such as irrigation and drainage lines become readily apparent due to the absence of inflorescence. Other areas of low density are also visible. Figure 9B exhibited average inflorescence as predicted by the model and confirmed by the horticulturalists. In Figure 9C, failed forcing was evident in two beds in the middle of the block. We saw that while most rows had successfully flowered at almost 98% fruiting, a single bed down almost half the length of the middle of the block showed poor forcing at only 62%. This lack of flowering was possibly due to either a blocked sprayer nozzle or incomplete overlap between the sprayer arms. Automatically and immediately identifying issues caused by equipment provides tremendous value to the grower so the issue does not become present in other regions of the field.

Location (Figure 9D) was predicted to have a below-average flowering rate, however, inspection showed that the rate was comparable to surrounding areas. Plants in this area were shorter than average, resulting in the early fruit being more easily obscured. Very evident from the model's output was the area indicated at Figure 9E, which appeared to be completely lacking inflorescence. Field inspection confirms this was indeed true: a sizable portion of this block had remained vegetative and failed to flower in this triangular area. A complete absence of flowering in a pattern like this at the end of this block as likely due to the spray rig running out of chemical as it approached the end of its run.

Horticulturists inspected the areas (Figures 9F,G) which the model predicted as low density. Ground inspection indicated that this region is in fact flowered more than the model predicted. Examination of the original imagery shows that this region of the image was blurry, likely due to an issue during mosaicking, resulting in an artificially low prediction from the model. This will be explored further in section 5.

## 5. Discussion

### 5.1. Impact on Specialty Crops

Modifying management practices with data on field conditions goes beyond reducing costs for the farmers. By identifying flowering plants at their earliest stages across entire fields, the application of chemicals can be precisely applied and limited in extent. By monitoring the progression of plant development across the field, harvest times can be optimized so that fruit are picked at their peak development, limiting waste and maximizing return. On-going work is considering the potential to predict marketable fruit and percentage of unharvested fruit because of small size; this possibility is supported by the variation in flowering densities predicted by our algorithm.

### 5.2. Active Learning and Uncertainty Sampling

In the present work we have embedded our model in an active learning framework to continually collect new annotations and repeatedly retrain the model for continual learning and improvement. While the capacity of neural networks is immense (Brown et al., 2020), training on an ever-growing amount of data can be computationally cumbersome and expensive. Therefore, it can be advantageous to (re)train the model only on the subset of data which is “challenging,” that is, near the decision-boundary. This is the motivation behind our selection criteria for sample prioritization.

The focus of this work was not to determine the most optimal data selection process, but to identify *an* approach that could be used to reduce annotator burden and improve model performance. Here we have exploited a subtlety of the framework by noticing that “more difficult” examples tend to produce less well defined peaks that are more likely to be dropped during the peak finding step. Use of techniques such as uncertainty or adversarial sampling can be employed to identify data that should be inspected for annotation and fed back to the model for retraining (Žliobaitė et al., 2013; Mayer and Timofte, 2020). Even though we are far from having too much annotated data for the current model, the incorporation of hard example mining techniques like those mentioned above are still useful for prioritizing which samples the annotators correct first; exploring these techniques is the focus on ongoing work.

### 5.3. Orthorectification and Mosaicking

All of the models in this work were trained on data from large, orthorectified, mosaicked images. Orthorectification is a central part of remote sensing analysis, particularly when involving agriculture, because it controls for the effects of image perspective and relief; agronomic indices based on ground reflectance values rely on these corrections. As such, traditional CV algorithms are largely dependent on the mosaicking and orthorectification process. However, deep learning approaches, like those used here, rely on learned, non-linear features involving shape/structure, and color. This enables them to be more robust to variations such as lighting/reflectance shifts and able to generalize to broader domains as opposed to relying on upstream algorithms to identify and/or control for these variations. Since mosaicking requires the program to identify key points for alignment, a very uniform field with high density planting, present challenges and may lead to blurriness in some assembled areas of a mosaic.

It is likely that the current model, trained on mosaicked-orthorectified images, would initially perform slightly less well on unseen, non-orthorectified imagery because that data is slightly out-of-domain. However, it is reasonable to believe that with minimal fine-tuning and retraining on such imagery, the model would perform equivalently well in the new domain; as humans, the task of identifying flowers from either sources is equivalent in difficulty and both tasks would be considered a “Type 1” process (Kahneman, 2011). Enabling inference directly on the raw imagery would cut out a time-consuming step of the processing pipeline and enable a wide range of live and on-device applications. As future work, we will examine the impact of working directly on raw images both from RGB and specific spectral bands and explore transfer-learning approaches to adapt the model to this new, but similar, domain.

Additionally, we saw in section 4.3 that the model performed less well on regions of the field which were fuzzy, potentially due to mosaicking issues. This is not surprising as degraded image quality would be expected to result in poorer performance. Nevertheless, as the inflorescence in this region are still discernible by humans from the fuzzy imagery, we believe that with additional annotation and training on degraded imagery, the model will be able to learn how to handle such sources of noise and generalize to a greater range of image quality.

### 5.4. Extension to Multiple Scales and Other Domains

All of the data here was flown at 200', producing images with similar statistical structure (i.e., all of the plants and inflorescence are roughly the same size). To make this algorithm broadly useful across many environments, we would like it to perform well across a variety of (reasonable) flight heights and resultant resolutions. Additionally, we would like to determine the minimum required resolution (i.e., maximum height flown) which delivers quality results; flying at a higher elevation would allow the data to be collected more rapidly.

Handling multiple scales is another place where deep learning shines over traditional computer vision algorithms. Flying at a given height allows the model to learn that inflorescence are all roughly the same size; flying at multiple elevations would require the model to learn a more expansive filter-bank to identify inflorescence of widely varying sizes. Although, we anticipate this transfer task to be more challenging than the one from orthorectified to non-orthorectified, we are confident that the model could generalize to handle multiple scales because of the successes of deep learning approaches in the broader crowd counting space. Should the current model struggle to handle multiple scales, there are a number of scale and context-aware modifications we could make in the current framework which would address these challenges (Hossain et al., 2019; Liu et al., 2019). Multi-scale detection in this domain is the focus of future work.

Similarly, this analysis was conducted on blocks from a single field under the same management conditions. Deep learning approaches again provide us with the ability to more easily adapt to unseen domains such as different fields under different management. Because these approaches do not rely on handcrafted rules and features but instead learn the relevant features directly from the data, knowing these management practices or appearance differences *a prior* is not necessary. Given the success of other deep learning models to generalize with increasing data, we believe the current model will generalize over a wide range of appearances, seasons, and management practices, particularly as we continue to supply it with new data efficiently obtained under the active learning paradigm.

### 5.5. Real-Time Edge Deployment and Alerting

A key advantage of this approach is speed of inference and lightness of the model architecture; not only is the model fast, but its performance is constant and does not degrade as the number of detected entities increases. Because a single image can be processed in <0.01 s on a GPU, this opens the possibility for real-time deployment. While the current model is trained and inferenced on a GPU, it has not yet been compiled for target hardware through an optimized runtime like TensorRT^1^, further increasing the inference speed. This would enable edge deployment: one could envision running the model live while a drone is collecting the imagery and providing alerts when encountering low-density flowering areas.

**Figure 10:**
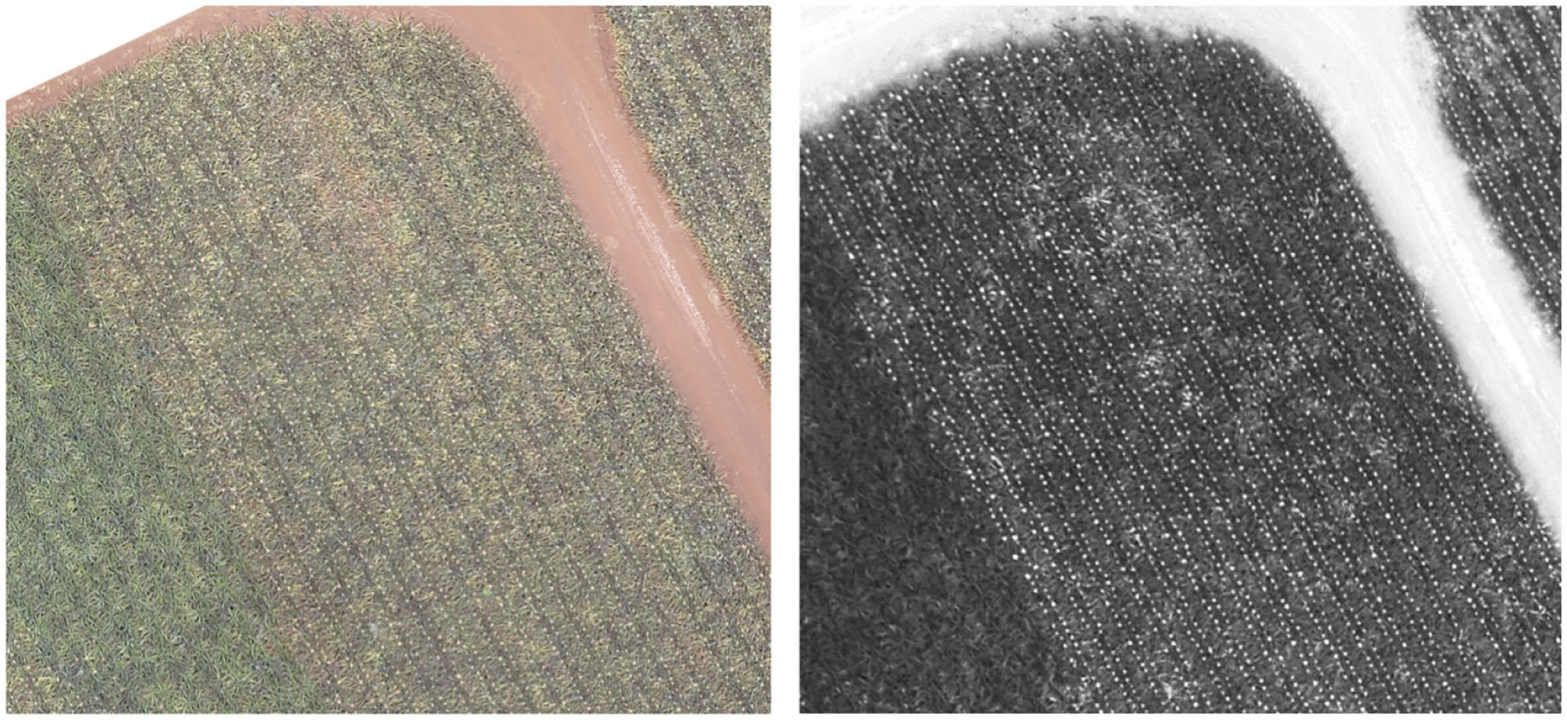
An image of the field taken with RGB **(Left)** and Red-Edge **(Right)**. The inflorescence are readily apparent in the single-channel red-edge image, suggesting this would be a useful addition in future analysis. This block highlights the potential of our system as the leftmost portion of this block in both images is still vegetative with no flowering.

The alerting component that this model enables, either real-time on the edge or after batch-processing, also has key value to growers. While the aerial imagery itself provides the growers with novel information not accessible from manual ground inspection (see Figure 10, left), most growers are not interested in or compelled by the raw imagery alone. Instead, most prefer to have an intelligence layer that sits on top of the imagery and alerts them to regions under their management requiring attention. Once the density map is determined by this current application, it can be handed off to a second application which identifies regions of the field which are anomalous or problematic and automatically alerts the growers accordingly; this is the focus of ongoing work.

### 5.6. Beyond RGB

Although not discussed in detail here, determining the right camera and flight height/resolution was an important step in the data acquisition process. The present analysis focuses only on RGB data as we were able to obtain very good results from the three-channel images. However, other channels may further improve model performance, stability, and generalization. Figure 10 shows a region of the field [corresponding to the area in Figure 9E] taken in RGB (left) and with a Red-Edge (right). The inflorescence visually “pop” in the red-edge image and are easily identifiable. Therefore, incorporating collecting additional red-edge imagery and training the model on a four-channel input could be very beneficial. Future work will explore incorporating additional channels like the red-edge seen here.

## 6. Conclusion

We have developed a density-estimation deep learning model based on a U-net backbone that accurately detects flowering pineapple plants in a field. Because of the architectural decisions made, the model is fast, lightweight, and its computational efficiency is independent of the number of inflorescence detected, allowing us to rapidly detected over 1.6 million flowering plants in a field. Our model highlights areas on the field which are vegetative or demonstrate failed forcing; growers can be alerted to these areas which would otherwise go undetected. Finally, the model will continue to improve as more corrected annotations are fed back into the model for retraining through our active learning system.

## Data Availability Statement

The datasets presented in this article are not readily available because, data may not be used for commercial purposes. Requests to access the datasets should be directed to Jennifer Hobbs, jennifer+research@intelinair.com.

## Author Contributions

PP, HH, and JH were involved in the model development. RP, BM, GR, and JH were involved in the data acquisition, annotation, and ground-truthing. JH took the lead in preparing the manuscript. All authors provided critical feedback and analysis.

## Conflict of Interest

JH, PP, HH, BM, and GR were employed by IntelinAir, Inc. The remaining author declares that the research was conducted in the absence of any commercial or financial relationships that could be construed as a potential conflict of interest.

## Footnotes

^1^https://developer.nvidia.com/tensorrt
