# Corrigendum: Large-Scale Counting and Localization of Pineapple Inflorescence Through Deep Density-Estimation

**DOI:** 10.3389/fpls.2021.695397

**Published:** 2021-05-17

**Authors:** Jennifer Hobbs, Prajwal Prakash, Robert Paull, Harutyun Hovhannisyan, Bernard Markowicz, Greg Rose

**Affiliations:** ^1^IntelinAir, Inc., Champaign, IL, United States; ^2^Department of Electrical Engineering, Columbia University, New York, NY, United States; ^3^Department of Tropical Plant and Soil Sciences, University of Hawaii at Manoa, Honolulu, HI, United States

**Keywords:** deep learning-artificial neural network (DL-ANN), active learning, pineapple, computer vision, remote sensing-GIS, weakly supervised, counting, density estimation

In the original article, there was a mistake in Figure 8 as published. The labels for the Validation and Test losses were switched in the legend. The Validation loss should be indicated as a red line with circular markers and the test loss should be indicated as a red line with starred markers. The text and caption described the figure correctly and therefore remain unchanged. The corrected Figure 8 appears below.

**Figure 8:**
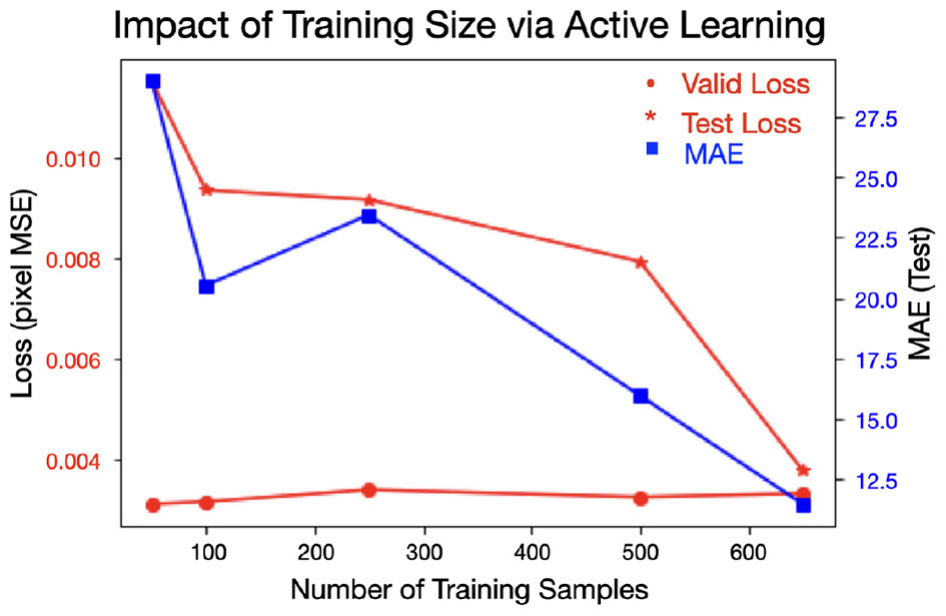
Increasing the amount of (labeled) training data in a smart fashion decreases test loss as well as the MAE on the test set. The validation loss slightly increases as more data is added, suggesting less over-fitting is occurring as more data is added.

The authors apologize for this error and state that this does not change the scientific conclusions of the article in any way. The original article has been updated.

